# Cost analysis of implementing community-based mass drug administration for schistosomiasis control among adult individuals using community drug distributors in Ukerewe district council, North-Western Tanzania

**DOI:** 10.1371/journal.pntd.0014470

**Published:** 2026-07-06

**Authors:** Jackson M. Bwimba, Alphoncina Kagaigai, Elizabeth Gudu, Irene Msirikale, Ruchius Philbert, Crecencia E. Chiombola, Charles Guya Mkombe, Saskia Kreibich, Christa Kasang, Antje Fuss, Andreas Mueller, Humphrey D. Mazigo, Amani Thomas Mori

**Affiliations:** 1 Department of Pharmacy, Muhimbili National Hospital (MNH), Dar es Salaam, Tanzania; 2 Department of Paediatrics and Child Health, Muhimbili National Hospital (MNH), Dar es Salaam, Tanzania; 3 School of Public Health and Social Sciences, Muhimbili University of Health and Allied Sciences (MUHAS), Dar es Salaam, Tanzania; 4 Department of Finance, Catholic University of Health and Allied Sciences, Mwanza, Tanzania; 5 Department of Medical Parasitology and Entomology, Catholic University of Health and Allied Sciences, Mwanza, Tanzania; 6 District Medical Department, Ukerewe District Council, Nansio, Ukerewe, Mwanza, Tanzania; 7 DAHW - German Leprosy and Tuberculosis Relief Association, Würzburg, Germany; 8 medmissio – Institute for Global Health, Würzburg, Germany; 9 University Hospital Würzburg, Medicine II, Infectious Diseases and Tropical Medicine, Würzburg, Germany; 10 University Hospital Würzburg, Medicine II, Internal Medicine-Hepatology Unit, Würzburg, Germany; 11 School of Public Health, Catholic University of Health and Allied Sciences, Mwanza, Tanzania; 12 Department of Global Public Health and Primary Care, Section for Ethics and Health Economics, University of Bergen (UiB), Bergen, Norway; Wellcome Sanger Institute, UNITED KINGDOM OF GREAT BRITAIN AND NORTHERN IRELAND

## Abstract

**Background:**

Schistosomiasis is a significant public health challenge in sub-Saharan Africa, with Tanzania having the second-highest disease burden. While mass drug administration (MDA) with praziquantel is effective, high-risk groups including adults living in remote rural areas are often neglected. To achieve schistosomiasis elimination by 2030, it is important to expand mass treatment to groups left out of interventions. The five-year Ukerewe Schistosomiasis Control Program has expanded the MDA campaigns to include the adult population through a community-based MDA implemented by community drug distributors in remote islands. This study aimed to estimate the implementation costs of the first phase of the Ukerewe Schistosomiasis Control Program among the adult population residing on Ukerewe Island, Tanzania.

**Methods:**

A descriptive cross-sectional study was conducted from a provider’s perspective, using a combination of activity-based costing and ingredients approaches to identify, quantify, and value all resources consumed in implementing the MDA. The analysis includes 30 villages and targeted 200,000 individuals. Secondary data were collected from financial records, inventory and transportation logs, and program budgets. Costs were categorized as capital if incurred on items that last more than a year or else as recurrent. Capital costs were annualized with a discount rate of 3% and annualization factor related to their useful life years (economic cost) or by straight-line depreciation method to estimate their economic and financial costs, respectively). Costs were collected in Tanzanian shillings (TZS) and adjusted to 2025 based on prevailing exchange rates from the Bank of Tanzania. The analysis was performed using an Excel spreadsheet (Microsoft Excel, Microsoft Corporation).

**Results:**

From 2021 to 2025, the economic and financial costs of the program were estimated at USD 377,189 and USD 208,999 respectively. This translates into an average economic and financial costs of USD 1.2 and USD 0.7 per person treated. Nearly three-quarters of the economic cost (70%) was attributed to drug distribution and treatment, followed by training, sensitization, and monitoring activities. Recurrent costs, primarily for medicines and personnel, accounted for 97% of total cost. Cost projections through 2030 indicate that the total MDA costs will rise with population growth; however, the unit cost is expected to decline, signaling economies of scale and improved cost-efficiency.

**Conclusion:**

The Ukerewe MDA program provides strong evidence that large-scale, cost-efficient schistosomiasis treatment is feasible with robust planning. Future program efficiency can be further enhanced through improved supply chain systems, bulk procurement, and consideration of lower-cost praziquantel biosimilars, offering a scalable model for schistosomiasis control in other high-burden regions.

## Author summary

Schistosomiasis is a parasitic disease that affects millions of people in sub-Saharan Africa, especially communities living near lakes and rivers. In Tanzania, mass drug administration (MDA) with praziquantel has been carried out since 2004, mostly targeting school-aged children. However, many high-risk adults, such as fishermen and farmers, remain untreated, allowing transmission to continue.

The Ukerewe Schistosomiasis Control Program was launched in 2021 to address this gap by extending treatment to adults in remote island communities on Lake Victoria. We analyzed the cost of implementing this program using data collected between 2021 and 2025. During this period, more than 310,000 people were treated, exceeding the initial target. The average economic cost was approximately 1 US dollar per person treated, with medicines and personnel accounting for the largest share of total costs.

These findings show that large-scale, community-based treatment is feasible and affordable. With stronger drug supply systems and integration into other health campaigns, this model could be scaled up across Tanzania and other endemic countries.

## Introduction

Schistosomiasis is caused by parasitic worms, primarily *Schistosoma haematobium* and *Schistosoma mansoni*, and affects over 240 million people globally. In sub-Saharan Africa alone, it accounts for over 90% of global cases and causes between 150,000 and 280,000 deaths each year [1]. After Nigeria, Tanzania ranks second in the region in terms of prevalence, with the disease burden concentrated along the Lake Victoria zone. Within this area, Ukerewe district council carries the highest burden of intestinal schistosomiasis. Residents frequently come in contact with infested water bodies through fishing, agriculture, bathing, and domestic chores, which perpetuates local transmission [2–4]. The parasite’s life cycle involves freshwater snails as intermediate hosts and humans as definitive hosts. Without treatment, chronic infections can lead to serious complications including kidney failure, bladder cancer, liver fibrosis, and increased susceptibility to HIV [5–7]. In addition to its ill-health consequences, the disease also imposes a heavy economic burden on individuals and household. Children often experience reduced school attendance and academic performance, while adults suffer productivity losses, compounded by out-of-pocket healthcare costs, which are challenges that are particularly significant in low income communities in Tanzania [8,9].

The World Health Organization (WHO) recommends preventive chemotherapy through MDA with praziquantel as a primary strategy for schistosomiasis control [10]. Tanzania initiated MDA in 2004, targeting school-aged children in high-risk areas [11]. While this approach reduced disease prevalence among children, it largely excluded high risk adult populations including fishermen, farmers, women, and children under five. These untreated groups remain major reservoirs of infection, undermining elimination efforts and continuing to endure the chronic manifestation of the disease [12,13]. To address this gap, the WHO has called for the expansion of MDA coverage to previously unreached geographical areas and communities, including neglected adults and pre-school aged children [14–17].

In response, the Ukerewe Schistosomiasis Control Program was implemented between 2021 and 2025 to expand MDA coverage to all high-risk groups in the lakeshore communities of Ukerewe Island, Mwanza. The initiative is a collaborative effort involving the Catholic University of Health and Allied Sciences/Bugando Medical Centre, DAHW German Leprosy and Tuberculosis Relief Association and Medical Mission Institute Würzburg, Germany. The interventions implemented under the program comprise annual MDA campaigns, which targeted teenagers from 15 years of age (who are not in primary schools) to adults, test-and-treat activities, community health education, ultrasound screening for chronic schistosomiasis-related morbidities, and the management of *Schistosoma mansoni*-induced hepatosplenic complications, including esophageal varices [13,18]. In addition, the program focuses on strengthening the capacity of health facilities to diagnose and manage schistosomiasis, while utilizing an established network of community drug distributors to ensure wide population coverage [13,18]. The initiative currently operates across 30 sentinel villages, covering a population of more than 200,000 individuals. Its overarching objectives are to reduce transmission, prevent long-term complications, and generate scalable models that can be adapted for implementation in other endemic regions [13,18]. Despite the considerable scope and ambition of this integrated approach, there remains a paucity of evidence on the economic costs associated with such large-scale MDA program. Understanding these costs is essential to inform resource allocation, guide strategic planning, and support the sustainability of Schistosomiasis control efforts. Accordingly, this study aimed to estimate the implementation costs of the first phase of the Ukerewe Schistosomiasis Control Program among the adult population residing on Ukerewe Island, Tanzania.

## Methods

### Ethical considerations

Ethical approval for this study was granted by the Muhimbili University of Health and Allied Sciences (MUHAS) Institutional Review Board (Ref: MUHAS-REC-03-2025-2771). Permission to access program data was obtained from the implementers of the Ukerewe Schistosomiasis Control Program. The costing study included only data on expenditures related to the implementation of the MDA campaign. Data collection did not involve human participants and therefore informed consent was not required. the costing data originated from the project which received ethical approval from the National Ethical Review Committee board (NIMR/HQ/R.8a/Vol.IX/3590 and NIMR/HQ/R.8C/Vol.I/1973). Written informed consent (>18 years) and written informed assent (15–17 years) were obtained from all participants [19,20].

### Study design and perspective

A retrospective cross-sectional study was conducted to estimate the financial and economic costs of the Ukerewe SchistosomiasisMDA program.

The analysis was undertaken from the **provider perspective**, capturing all the costs incurred by the program for in the delivery and management of MDA activities.

### Costing approach

We used a combination of activity-cased Costing and ingredients approaches. Costs were initially categorized by activity type, including community sensitization, training (for Community Drug Distributors, healthcare workers and community leaders), annual implementation of community-based MDA and monitoring and evaluation. This was followed by the identification and quantification of all resources consumed by each activity.

The resources were assigned monetary values as reflected in the financial records such as receipts and invoices. Costs for each activity were aggregated, ultimately leading to a summary of the total program costs.

### Economic vs financial costs

Economic and financial costs were defined to distinguish between actual expenditures and the full value of resources used. Financial costs represent all direct monetary outlays incurred during program implementation and exclude donated resources such as medicines. Economic costs, on the other hand, capture the total value of all resources utilized, including the opportunity cost of donated goods and services [21–23].

In this study, financial costs were estimated by capturing all expenditures related to MDA implementation excluding donated resources such as medicines, while economic costs were derived by incorporating the value of donated inputs and other non-financial resources to provide a more comprehensive estimate of program costs.

**Capital costs** are one-time investments in assets or infrastructure that have a useful life of more than one year. This included central-level expenditures on vehicles, medical equipment, computers, and office furniture. These costs were estimated using a Straight straight-line depreciation method over their useful life years to estimate their financial costs, while Economic costs were calculated by annualizing capital costs on the basis of a 3% discount rate in line with the WHO guidelines [24].

**Recurrent costs** are ongoing expenditures on items that have a useful life of less than one year, such as staff salaries, supplies, utilities, office maintenance, and other operating expenses. Personnel costs were allocated based on the proportion of time spent on program activities. Additionally, expenses for training activities, monitoring and evaluation, as well as the costs of praziquantel and other consumables, were thoroughly documented and apportioned to specific program components such as drug distribution and capacity building.

Where data inconsistencies or gaps were identified, clarification was sought from the Principal Investigator, Program Manager, and Program Accountant to validate and refine the cost information.

For the estimation of economic costs, donated items were valued at their **market price** to capture the opportunity cost of the resources utilized in the program. In this study, the drugs (praziquantel) donated by the **World Health Organization** were distributed by the **Ministry of Health** to district hospitals then to nearby health centers based on identified needs, where community drug distributors collected them for MDA activities. The donated drugs were assigned a monetary value based on the corresponding unit prices listed in the **Medical Stores Department (MSD) price catalogue** for each respective year from **2021 to 2025.**

### Study setting, study population and sample size

**Fig 1:**
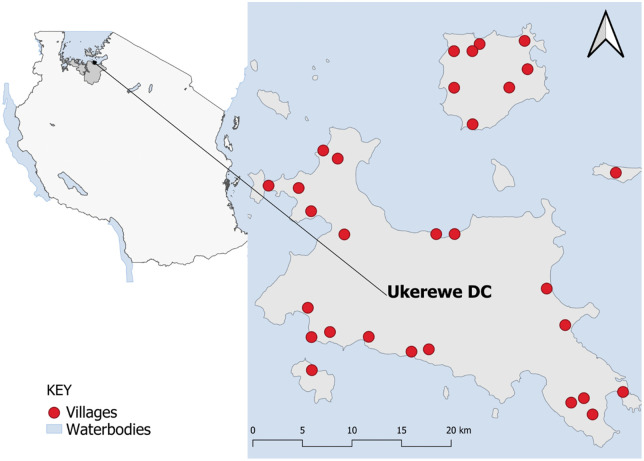
Map of 30 villages in Ukerewe District covered by the Schistosomiasis Control Program (credit to [href:https://gadm.org/maps/TZA_1.html]https://gadm.org/maps/TZA_1.html).

Ukerewe District, located in Mwanza Region in northwestern Tanzania, comprises 38 islands on Lake Victoria, 15 of which are permanently inhabited. The district has a population of 387,815 (2022 census), primarily dependent on subsistence farming, fishing, and small-scale trade. Health services are provided through one district hospital, four health centers, and 32 dispensaries. The MDA program targeted individuals (15 years and above) across 30 villages on Ukerewe Island, which is highly endemic for intestinal schistosomiasis (Fig 1). The target population comprised adult community members not covered by the routine school based MDA program, including adolescents aged 15–17 years who were not enrolled in school. School-going children were excluded, as they received preventive chemotherapy through routine school-based MDA interventions implemented separately.

This study utilized secondary data from the Ukerewe Schistosomiasis Control Program to estimate the costs incurred during MDA implementation. The program targeted approximately 40,000 community members annually through MDA campaigns, with a projected total of 200,000 individuals treated over the five-year (2021–2025) implementation period and the data for the age groups 15–17 years covered by the program were pooled together with the rest of the population. A census approach was employed for the costing analysis, whereby all available expenditure and financial records related to MDA implementation during the study period were included.

### Time horizon

This study analyzed the costs incurred over five years for the Ukerewe Schistosomiasis Control Program, which was launched in February 2021 and is scheduled to run through 2030. The cost analysis was specifically focused on activities implemented up to May 2025, signifying the completion of the program’s initial phase.

### Data collection methods

#### Data collection.

Secondary data was collected from program’s financial records, including invoices, receipts, transport logs, and payrolls. The **Tool for Integrated Planning and Costing (TIPAC)**, developed by RTI International and USAID, was used to design the data collection tool for this study and to structure data entry and categorize expenditure according to standard NTD program cost classifications [16,25].

The costs data were organized according to the activity roadmap outlined in the MDA implementation protocol, which grouped program activities into four primary cost centers: **community sensitization**, **training**, **drug distribution and treatment**, and **monitoring and evaluation of coverage**. This structured framework facilitated a systematic assessment of program expenditures and enhanced the accuracy and efficiency of resource allocation across implementation components. Costs were further broken down into capital and recurrent costs, to furthercharacterize the nature of resources used.

### Data management and statistical analysis

All cost data were entered and analyzed using Microsoft Excel. Costs were summarized by activity, input type, and cost category (capital vs. recurrent). The unit cost per person treated was calculated by dividing the total annual program cost by the number of individuals who received praziquantel in each implementation year. The average unit cost over the five years was obtained by dividing the cumulative program cost by the total number of individuals treated during the same period.

Cost data were collected in Tanzanian Shillings (TZS) and adjusted to 2025 values using the Consumer Price Index (CPI) for the respective years to account for inflation. The inflation adjusted costs were then converted to U.S. Dollars (USD) using the Bank of Tanzania’s exchange rate of 2,615.6 TZS per USD, as of June 18, 2025.

Sensitivity analysis was conducted to identify key cost drivers for MDA implementation by varying major cost components by ±20%. In addition, cost projections for national MDA scale up through 2030 were developed using the 2022 population census as the baseline and applying an annual population growth rate of 2.5%. These projections estimated expected trends in total and per-person MDA costs under the assumption of sustained full population coverage each year.

## Results

### Treatment coverage

The first phase of the Ukerewe MDA program aimed to reach 40,000 community members annually from 2021 to 2025. The target was to reach a total of 200,000 individuals aged 15 years (who are not in primary school) and above across the island. The number of individuals who received praziquantel varied considerably over the implementation period, ranging from 32,071 in 2021–117,382 in 2025. No MDA was conducted in 2022 due to a drug stock-out. Cumulatively, the program reached 310,807 individuals, representing a 55.4% increase above the initial target. This overachievement was largely due to the program compensating for the missed MDA in 2022. Also in 2023, the program supported the Ukerewe District Council in expanding MDA delivery to primary school children, a group that was originally outside the program’s scope.

### Cost profile for the first phase of MDA

The financial cost and Economic cost for the Ukerewe MDA program varied significantly over the five years of implementation period, reflecting changes in program scale, drug availability, and operational intensity. In 2021, the economic cost and financial cost were USD 80,254, and USD 64,018 respectively, reflecting the initial rollout of the program. In 2022, the financial costs dropped sharply to USD 12,049 due to the suspension of MDA activities caused by a praziquantel stock-out, with spending primarily limited to preparatory and operational overheads. The highest economic costwas recorded in 2023 in the tune of USD 126,815, while financial cost USD 65,123 corresponding with a major treatment campaign that reached over 90,000 individuals. In 2024, the economic cost dropped further toUSD 80,502, and the financial cost to USD 43,639. In 2025, the economic cost and financial costs werewas USD 77,569 and USD 24,170, respectively. Overall, the total economic and financial costs of implementing the Ukerewe Schistosomiasis MDA program over the five years amounted to USD 377,189 and USD 208,999, respectively. This translates into economic and financial cost ofUSD 1.2 and USD 0.7 per person treated, respectively. The fluctuation in annual costs reflects changes in implementation scale, drug availability, and the evolving scope of the program as illustrated in Table 1.

**Table 1:** Cost incurred across all activities of MDA since 2021–2025 in USD.

Activities	2021		2022		2023		2024		2025		Total cost	
	Financial	Economic	Financial	Economic	Financial	Economic	Financial	Economic	Financial	Economic	Financial	Economic
Sensitization	7,074	7,074	7,703	7,703	13,138	13,138	10,100	10,100	5,709	5,709	43,724	43,724
Training	22,610	22,610	4,346	4,346	10,663	10,663	6,700	6,700	0	0	44,319	44,319
Purchase of drugs	–	16,235	–	–	–	61,692	–	36,863	–	53,399	–	168,189
Drug distribution and treatment	24,795	24,795	–	–	25,921	25,921	24,010	24,010	18,461	18,461	93,187	93,187
Monitoring & Evaluation	9,540	9,540		–	15,401	15,401	2,829	2,829	–	–	27,770	27,770
Total cost	**64,018**	**80,254**	**12,049**	**12,049**	**65,123**	**126,815**	**43,639**	**80,502**	**24,170**	**77,569**	**208,999**	**377,189**
No. of people treated	32,071		0		90,105		71,249		117,382		310,807	
Cost per person treated	2.0	2.5	–	–	0.7	1.4	0.6	1.1		0.7	0.7	1.2

### Cost associated with each MDA activity

Among the four core MDA activities, drug distribution and treatment accounted for the largest share of total economic cost amounting to USD 261,376 or 69.3% of the total cost. Of this, the cost of donated medicines constituted USD 168,189, representing 64.3% of the total cost for this activity, while the remaining USD 93,187 (35.7%) was attributed to logistics and personnel involved in implementation.

This was followed by training at USD 44,319 (11.7%), sensitization at USD 43,724 (11.6%), and monitoring and evaluation at USD 27,770 (7.4%). These figures clearly show that medicines cost, its distribution and treatment required the greatest financial investment, as illustrated in Table 1.

Overall, **recurrent economic costs** were much higher than capital costs, accounting for **USD 357,226,** equivalent to **94.7%** of the total program economic cost**.** Within the recurrent category, medicines accounted for **47.1%** (**USD 168,091)** and **personnel** 44% (**USD 157,344**).

The sensitivity analysis demonstrated that varying medicine and personnel costs by ±20% significantly influenced total MDA economic costs. A 20% reduction in either cost component yielded approximately 10% lower total program costs, while a 20% increase resulted in approximately more than 10% higher expenditures. These findings highlight the substantial weight of medicine and personnel costs in the overall budget structure.

**Fig 2:**
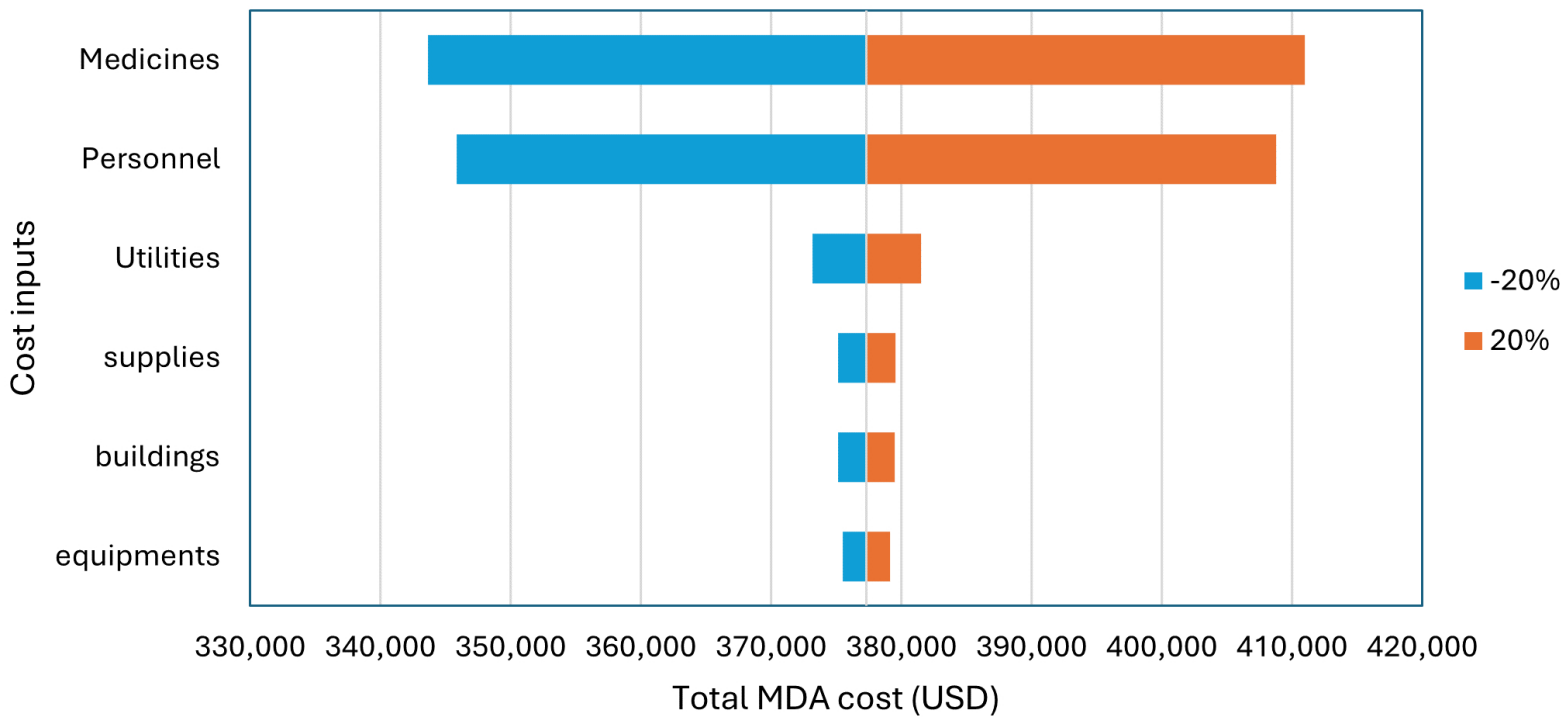
Tornado diagram showing key economic cost drivers of MDA implementation.

In contrast, capital investments such as equipment and building-related costs were minimal. These findings underscore that the primary financial burden of the MDA program was driven by recurring, service-delivery-related expenses rather than infrastructure or equipment, as shown in Fig 2.

### Cost projection for MDA expansion

As part of the efforts to eliminate schistosomiasis across Ukerewe Island and beyond, the goal of the program was to achieve full population coverage. According to the 2022 national census, the island’s population was 387,815. To support planning for scale-up, projections were made for the total and per-person MDA costs from 2022 through 2030, using an annual population growth rate of 2.5% forUkerewe district as per the 2022 census.

**Fig 3:**
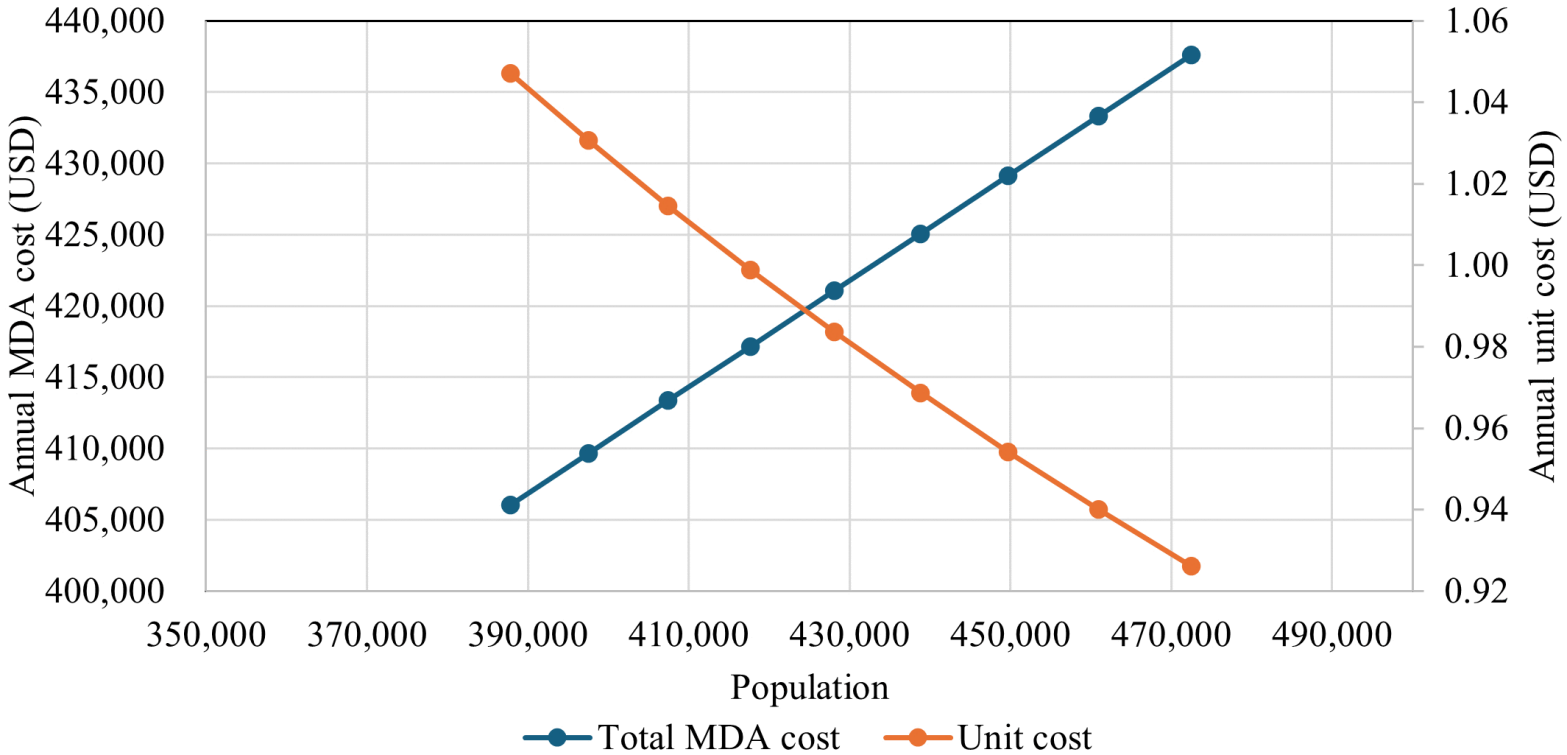
Projected Trends in Total and Unit economic Costs of MDA for Schistosomiasis control With Increasing Population in Ukerewe (2022–2030).

The projected total annual economic cost of MDA increases steadily with population growth, rising from USD 406,039 in 2022 to USD 437,612 by 2030. Conversely, the **cost per person** declines over the same period, from USD 1.05 in 2022 to USD 0.93 in 2030 as shown in Fig 3.

## Discussion

Schistosomiasis, a parasitic disease second only to malaria in its public health impact, remains highly endemic in Sub-Saharan Africa. Both urogenital and intestinal forms of the disease are prevalent, and Tanzania ranks among the most affected countries [2]. MDA using praziquantel has been the cornerstone strategy for schistosomiasis control [26]. However, for sustained impact, it is critical to understand the cost implications of such programs to better plan for future expenditure, improve program efficiency, and support expansion to other endemic areas [3]. The findings from the UkereweMDA program provide important insights into both the successes and operational challenges of implementing large-scale disease control interventions in high-burden settings. From 2021 to 2025, the program set out to reach 200,000 indiviaduals with praziquantel treatment across Ukerewe Island. By the end of the five years, the program had treated a total of 310,807 individuals, exceeding its original target by 55.4%. This increase can be attributed to the program strategies to expand its geographical coverage from the initial 15 villages to 35 villages and the government support to ensure the MDA drugs were available to cover the additional targeted population.

Despite this success in reaching more people, implementation was not without challenges. Notably, in 2022, no MDA treatments were administered due to a stock-out of praziquantel, despite the program incurring operational costs for preparatory activities such as training and sensitization. This reflects a common challenge in MDA programs across sub-Saharan Africa, where delays in procurement and weak supply chains often interrupt service delivery. Similar findings have been reported in Uganda, emphasizing the need for robust supply chain systems and effective contingency planning to ensure continuity of services even in the face of logistical challenges [27].

Over the five year implementation period the program incured a total financial cost of USD 208,999 while the corresponding economic cost accounting for the value of donated medicines amounted to a USD 377,189. The annual costs fluctuated with the scale and intensity of implementation. The highest economic cost was recorded in 2023, which coincided with a large-scale treatment campaign reaching over 90,000 individuals. In contrast, the year 2022 saw the lowest cost of USD 12,049, due to the absence of treatment activities, highlighting inefficiencies from fixed costs incurred without service delivery.

Analysis of unit cost trends over the five-year data reveals consistent improvements in cost efficiency, with economic costs per person treated declining from USD 2.5 in 2021 to USD 0.70 in 2025 as treatment numbers increased, demonstrating an economy of scale. This pattern mirrors findings from other programs elsewhere, including Uganda where per-child treatment costs dropped from USD 0.91 to USD 0.41 (with delivery costs falling from USD 0.69 to USD 0.19) as coverage expanded from 7,161–37,032 children [27], and South Africa where despite rising medication expenses, unit costs decreased by 19% (to R110.72) at 50% coverage and by 34% (to R90.77) at 90% coverage [28]. The higher unit cost observed in 2021 can also be attributed to substantial initial investments in purchasing equipment and furniture, which elevated start-up costs during the first year of implementation. These consistent cross-country trends confirm that scaling up treatment programs not only extends population coverage but also significantly improves cost efficiency through better amortization of fixed costs across larger beneficiary groups.

A closer look at the cost structure of the Ukerewe MDA reveals that medicine costs, their distribution and treatment activities were the most resource-intensive, consuming 69.3% of the total economic cost. Training and sensitization each accounted for about 12%, while monitoring and evaluation represented 6.9%. Recurrent costs dominated the budget (94.7%), primarily comprising medicines (praziquantel, 47.1%) and personnel costs (44%), which accounted for 86.3% of the total costs. Capital investments were minimal contributing just 5.3% of the total program cost. These findings align with systematic review evidence on schistosomiasis control costs, which showed similar patterns: drugs represented the largest expenditure category (49%), followed by personnel and training (21%), operational costs (18%), materials/equipment (9%), diagnostic tests (18% where applicable), behavioral interventions (10% where present), and miscellaneous costs (2%) [29]. These findings are also consistent with the studies done in Niger, Burkina Faso and Uganda [30].

Looking ahead, the study developed cost projections to estimate the financial implications of scaling up MDA to achieve full population coverage. Using the 2022 national census data and a 2.5% annual population growth rate [31], projections showed that the total MDA economic cost cost would gradually increase from USD 406,039 in 2022 to USD 437,612 by 2030. However, the cost per person treated would continue to decline, from **USD 1.05** in **2022** to **USD 0.93** in **2030**. These trends suggest that scaling up MDA is both feasible and increasingly cost-efficient, especially if implementation remains consistent and uninterrupted. Similar projections have been validated in Sierra Leone and Ethiopia, where expanded treatment coverage led to lower per capita costs due to improved logistics, shared resources, and sustained community mobilization [32].

Despite the efficiency gains associated with scaling up MDA in Ukerewe, the projected unit costs for scaling up MDA remain higher than the US$ 0.20 reported in Zanzibar and above the commonly cited benchmark of less than US$ 0.50 per person treated for large-scale NTD programs. However, these differences should be interpreted within context. The estimate from Zanzibar excluded the cost of praziquantel while the benchmark assumes programs benefit from donated medicines and rely largely on local volunteer personnel [33,34]. In contrast, our study shows that medicines accounted for 47.1% of the total economic costs in Ukerewe. Additional differences may also arise from variations in logistical demands, personnel requirements, and early capital investments associated with program implementation. These considerations highlight the importance of clearly accounting for both financial and economic costs including donated commodities, volunteered time and delivery platforms when estimating and comparing unit costs across settings.The overall findings from the Ukerewe MDA program reinforce several important lessons. First, a consistent and reliable drug supply is key for achieving treatment targets and optimizing financial efficiency. Second, higher treatment coverage not only meets public health objectives but also reduces unit costs, strengthening the case for expansion. Third, the largest economic burden stems from recurrent cost items, particularly medicines and personnel, suggesting that cost-reduction strategies should prioritize optimizing supply chain systems and workforce efficiency over capital investments. Lastly, integrating MDA with other public health campaigns, such as deworming and neglected tropical disease (NTD) control programs and the use of primary healthcare facilities as points of distributing drugs, could enhance both reach and cost-effectiveness by leveraging shared platforms and resources. This approach aligns with findings from a study on the cost-effectiveness of Triple Drug Administration (TDA) using praziquantel, ivermectin, and albendazole for NTD prevention in Nigeria [35].

In line with the WHO targets of reaching at least 75% of school-aged children in endemic areas, the experience in Ukerewe presents a promising model for expanding MDA programs across Tanzania and similar settings. With improved logistics, integration, and monitoring, future phases of implementation can achieve greater impact while maintaining financial sustainability.

However, this study also faced challenges of inconsistently documented cost data and overlapping expenditures across different program activities. To address this, a structured cost categorization and allocation framework was applied, guided by clear procedures to ensure accurate cost attribution and minimize the risk of double-counting. This methodological rigor contributed to the reliability and validity of the overall cost analysis.

## Conclusion

The Ukerewe MDA program provides strong evidence that large-scale treatment for schistosomiasis is feasible when well-planned and supported. By treating over 310,000 people at a unit cost of about USD 1 far exceeded its original goal, enabling the program not only to improve access to essential medicines but also to show that expanding coverage helps reduce the cost of treatment per person. However, challenges such as drug stock-outs revealed weaknesses in the supply system that need urgent attention. Looking ahead, further savings could be made through bulk purchasing of medicines and exploring safe, lower-cost alternatives like praziquantel biosimilars. With better planning, stronger logistics, and continued investment, the Ukerewe experience can serve as a powerful model for expanding schistosomiasis control in other high-need areas of Tanzania and beyond.

## Recommendation

### To the Ukerewe Schistosomiasis control program

- Maintain disaggregated financial records for each program activity to support accurate cost evaluation, accountability, and better planning.
- Expand MDA coverage to more individuals to reduce unit costs and improve overall cost-efficiency through economies of scale.
- Strengthen coordination for drug supply by improving forecasting, establishing buffer stocks to prevent future stock-outs.
- Document and share lessons learned to inform future interventions and guide scale-up in other affected areas.

### To the Directorate of Preventive Services (DPS), Ministry of Health, which houses the NTD Program

- Strengthen coordination with the Medical Stores Department (MSD) to ensure a reliable supply of praziquantel by improving forecasting, planning, and distribution logistics, also establishing contingency mechanisms such as emergency stock reserves to mitigate potential supply chain disruptions.
- Integrate schistosomiasis MDA with other public health programs such as school deworming to improve efficiency by leveraging shared infrastructure and resources.
- Sustain high treatment coverage by targeting both school-aged children and other at-risk populations to maximize health impact and reduce unit costs through economies of scale.
- Institutionalize proper financial data management, including systematic archiving of disaggregated cost data, to support accurate cost analysis and inform future budgeting and planning.

### Study limitations and mitigation

This study utilized retrospective administrative data from the Ukerewe Schistosomiasis Control Program. Because expenditures were shared across multiple interventions and activities within the program, additional effort was required to identify the resources used and appropriately reclassify budget data so that costs could be attributed to MDA and to its specific components to minimize the risk of double-counting, enhancing the reliability of the analysis.

## Supporting information

S1 Data MDA USCP financial and economic data 2021.xlsx (XLSX)

S2 Data MDA USCP financial and economic data 2022.xlsx (XLSX)

S3 Data MDA USCP financial and economic data 2023.xlsx (XLSX)

S4 Data MDA USCP financial and economic data 2024.xlsx (XLSX)

S5 Data MDA USCP financial and economic data2025.xlsx (XLSX)

## Abbreviations

CYN: Chinese Yuan
CUHAS: Catholic University of Health and Allied Sciences
DALYs: Disability-Adjusted Life Years
HIV: Human Immunodeficiency Virus
ICER: Incremental Cost-Effectiveness Ratio
MDA: Mass Drug Administration
NTD: Neglected Tropical Diseases
PC: Preventive Chemotherapy
PZQ: Praziquantel
SAC: School Aged Children
TIPAC: Tool for Integrated Planning and Costing
USAID: United States Agency for International Development
WHA: World Health Assembly
WHO: World Health Organization
WSH: Water, Sanitation and Health education
ZAR: South African rand
