# Supplementary material for: Cost analysis of implementing community-based mass drug administration for schistosomiasis control among adult individuals using community drug distributors in Ukerewe district council, North-Western Tanzania
Source: PLoS Negl Trop Dis. 2026 Jul 6;20(7):e0014470. doi: 10.1371/journal.pntd.0014470 (PMC13354067; doi:10.1371/journal.pntd.0014470)
Supplement: Attachment — Submitted filename: Assessment report for the manuscript PNTD-D-25-02153.docx [file pntd.0014470.s006.docx]

**Assessment report for the manuscript titled Cost analysis of implementing community-based mass drug administration for schistosomiasis control among adult individuals using community drug distributors in Ukerewe district council, North-Western Tanzania**

Manuscript Number: PNTD-D-25-02153

1. Page 3, Author Summary, line 4: insert space between **adultssuch**

2. page 5, Introduction, line 14: insert space between **lowincome**

3. page 5, Introduction, line 18: insert space between **highrisk**

4. page 8, Cost approach, line 4: insert hyphen between **communitybased**

5. page 8, Cost approach, line 6: correct typo **susch**

6. page 8, Study setting, line 2: Why not use a more recent census estimate or projection since the growth rate is available? This might show the true difference in the number of people reached (received praziquantel) in 2025 (310,807 2022 census versus 387,815 that were reached at the end of 2025). Study population and sample size not clearly stated.

7. Page 10, Cost data Collection, line 18: Correct typo **methothod**

8. Page 11, Data Management, line 8 and 12: insert space between **inflationadjusted and scaleup**

9. Page 12, Treatment Coverage, line 1: who exactly are the target of the MDA programme? All the community members or Non-school children?

10. Page 12, Treatment Coverage, line 6: What was the extent of the expansion? Was the expansion within Ukerewe district/ Island/ MDA programme?

11. Page 12, Cost profile for the first phase of MDA, line 7: Judging by Table 1, the cost by year is not **~~TZS~~** USD 80,502

12. Page 14, Cost associated with each MDA activity, line 4: Judging by Table 1, the cost by year is not **~~TZS~~** USD 27,770

13. Page 15, Cost projection for MDA expansion, line 2: The District 2022 census estimate stated under study setting (387,815) is different from the one stated here (202,389). The two should have been stated and clarified under the study setting.

14. Page 17, Discussion, line 27 and 37: insert space between **costefficiency**
