# Supplementary material for: Cost analysis of implementing community-based mass drug administration for schistosomiasis control among adult individuals using community drug distributors in Ukerewe district council, North-Western Tanzania
Source: PLoS Negl Trop Dis. 2026 Jul 6;20(7):e0014470. doi: 10.1371/journal.pntd.0014470 (PMC13354067; doi:10.1371/journal.pntd.0014470)
Supplement: Attachment — Submitted filename: Response to reviewers .docx [file pntd.0014470.s009.docx]

PLOS Neglected Tropical Diseases

Dear editor,

Re: Resubmission of manuscript titled “Cost **analysis of implementing community-based mass drug administration for schistosomiasis control among adult individuals using community drug distributors in Ukerewe district council, North-Western Tanzania**”

We are grateful for being another opportunity to resubmit our manuscript for publication in your journal. We thank the reviewers for raising constructive comments to improve the content and quality of the manuscript. We believe we have addressed all of them to their satisfaction, however, we are happy to do more revision if deemed necessary.

Below are point by point responses to the raised comments

**RESPONSE TO JOURNAL REQUIREMENTS COMMENTS**

| **S/N** | **Section** | **Comments from the reviewer** | **PI’s response** | **Page No. where changes have been made** |
| --- | --- | --- | --- | --- |
| 1 | Author contributions | Please ensure that the CRediT author contributions listed for every co-author are completed accurately and in full | Corrected: Amani Thomas Mori | Page 1 |
| 2 | copyright or trademark symbols violation | We do not publish any copyright or trademark symbols that usually accompany proprietary names, eg ©, ®, or TM (e.g. next to drug or reagent names). Therefore, please remove all instances of trademark/copyright symbols throughout the text, including: - ® on page: 2 | Changed Microsoft Excel® to Microsoft Excel | Page 3 |
| 3 | Figures format | Please upload all main figures as separate Figure files in .tif or .eps format. For more information about how to convert and format your figure files please see our guidelines:  https://journals.plos.org/plosntds/s/figures | Changed to tif | Page 12,19 and 21 |

**RESPONSE TO REVIEWER 2 COMMENTS**

| **S/N** | **Section** | **Comments from the reviewer** | **PI’s response** | **Page No. where changes have been made** |
| --- | --- | --- | --- | --- |
| 1 | Author Summary | line 4: insert space between adultssuch | Typo corrected | Page 4,  line 4 |
| 2 | Introduction | line 14: insert space between lowincome  line 18: insert space between highrisk | Typo corrected | page 6  Line 14 and 18 |
| 3 | Cost approach | line 4: insert hyphen between communitybased  line 6: correct typo susch | The hyphen is already there. It appears there may be a formatting issue with hyphens in the version of the document the reviewer is using. Even in the sections above where I was asked to insert a space, there are hyphens in my document  Typo in line 6 corrected | page 9,  Line 6 |
| 4 | Study setting, Study population and sample size | line 2: Why not use a more recent census estimate or projection since the growth rate is available? This might show the true difference in the number of people reached (received praziquantel) in 2025 (310,807 2022 census versus 387,815 that were reached at the end of 2025).  Study population and sample size not clearly stated. | Projections were made for the period of 2022 to **2030** to provide evidence to inform the second phase of the project (July 2025–2030) for implementers to use in planning of the program expansion. | page 10 |
|  |  |  | Study population and sample size clarified | Page 11  Line 3 to 8 |
| 5 | Cost data Collection | line 18: Correct typo methothod | Typo corrected and we moved this part under “Costing approach” section | Page 9,  Line 14 |
| 6 | Data Management | line 8 and 12: insert space between inflationadjusted and scaleup | Changed from hyphen to space | Page 14,  Line 8 and 12 |
| 7 | Treatment Coverage | line 1: who exactly are the target of the MDA programme? All the community members or Non-school children? | The target population was individuals aged 15 years (who are not in primary schools ) and above | Page 14  Line 3 |
| 8 | Treatment Coverage, | line 6: What was the extent of the expansion? Was the expansion within Ukerewe district/ Island/ MDA programme? | The expansion was in Ukerewe district | Page 15,  Line 9 to 10 |
| 9 | Cost profile for the first phase of MDA | line 7: Judging by Table 1, the cost by year is not TZS USD 80,502 | TZS removed | Page 15  Line 10 |
| 10 | Cost associated with each MDA activity | line 4: Judging by Table 1, the cost by year is not TZS USD 27,770 | TZS removed | Page 18,  Line 7 |
| 11 | Discussion | line 27 and 37: insert space between costefficiency | Hyphen removed, space inserted | Page 22, Line 30 and page 23 line 10 |
|  | Results, Cost projection for MDA expansion | line 2: The District 2022 census estimate stated under study setting (387,815) is different from the one stated here (202,389). The two should have been stated and clarified under the study setting | baseline population changed to 2022 census data, I had mistakenly used the one of 2012 census | Page 19  Line 3 |

**SUMMARY AND GENERAL COMMENTS**

| **S/N** | **Section** | **Comments from the reviewer** | **PI’s response** | **Page No. where changes have been made** |
| --- | --- | --- | --- | --- |
| **1** | **Data collection methods** | My most important comments relates to economic vs financial costs. You mention that both were collected in the methods but that is all. I believe that both financial and economic costs should be clearly presented and that further information in the methods is needed to explain how you estimated the opportunity cost of resources that did not have a budget line item, or which were donated? | Added details of Financial cost and Economic cost, also in table 1 I have clearly indicated them by also adding the cost of medicines which were donated.  Included the calculation of Economic costs by annualizing capital costs on the basis of a 3% discount rate and also explained how we computed costs for donated praziquantel. | **Page 9**  **Line 9-18**  **And**  **Page 10**  **Line 12- 18** |
| **2** | **Data collection methods** | For Capital costs. Straight line depreciation method is appropriate for financial costs but not appropriate for economic costs. See the following paper for further guidance: https://doi.org/10.1093/heapol/17.1.112 | Included the calculation of Economic costs by annualizing capital costs on the basis of a 3% discount rate and also explained how we computed costs for donated praziquantel. | **Page 9**  **Line 20-21 and page 10 line 1** |
| **3** | introduction | you refer to “The initiative currently operates across 30 sentinel villages”. Apologies if this is my misunderstanding, but is this referring to an initiative that falls under the Tanzania SCH program, or the Ukerewe Schistosomiasis Control Program? The current wording implies that it is the whole program, as Ukerewe is not really mentioned until the end of the paragraph, and Tanzania as a whole is what is talked about in the preceding paragraph. Please clarify and modify the text accordingly. Related to this, having more background on the Ukerewe MDA program in the introduction would be beneficial, such as whether it only targets adults or SAC as well? | Added three sentences to add more clarity to this | Page 7  Line 6 to 10 |
| **4** | **Methods** | The order of the methods can be hard to follow. I suggest adding what you have under the Time horizon subsection to the Study design and perspective subsection. | **Rearranged as suggested** | **Page 11** |
| **5** | **Results and Discussion** | The phase non-school children can be confusing in this context. It is a reference to adult children not in school. More generally, the age groups treated need further clarification. | Non-school children wording removed  The target population was individuals from 15 years of age and above | **Page 14**  **Line 2**  **Page 22**  **Line 1** |
| **6** | Discussion | It would be helpful to have a few sentences comparing the magnitude of the MDA cost found ($1.66 per treatment) to other MDA cost estimates in more detail (particularly in Tanzania). The benchmark of $0.5 is still used but could be out of date. | Sentences have been added  and the data here have been revised on projection changed due to a using of 2022 census population to do projections | **Page 24**  **Line 11 to 22** |
|  |  | Please clarify how the drugs were distributed – community volunteers or health workers ? If community volunteers how where they valued. | This is mentioned in the introduction **community-selected drug distributors** were engaged in the program and received daily allowances paid by the program. These payments were valued and categorized as **personnel costs** in the costing analysis. | **Page 7**  **Line 16-17** |
|  |  | The authors should consider a limitations section. | Limitation section added | **Page 27** |
|  | Abstract methods | there's an extra space in front of one of the full stops. | corrected | **Page 3**  **Line 17** |
|  | Whole document | In several places, there is a miss space before a reference bracket e.g. Page 5 – Missing space before [11] “areas[11]”. | corrected | Whole document |
|  | Introduction | mass drug administration is spelled out again at the beginning of second paragraph. Can just use MDA here | corrected | Page 6,7,8 |
|  | Study design and perspective | Methods “provider perspective, capturing all the expenses of resources consumed in implementing MDA activities”. Saying “capturing all the expenses of resources”. sounds more like the societal perspective” I would modify the text to make the implications of the perspective clearer. | Wording changed to reflect provider perspective. “The analysis was undertaken from the provider perspective, capturing all the costs incurred by the program for the delivery and management of MDA activities.” | Page 8  Line 3 to 5 |
|  | Cost data Collection | methothod typo (method) | corrected | Page 9  Line 22 |
|  | Results and Discussion | Results Cost associated with each MDA activity. Delete (M&E) as you do not use it. Check why you have the “TZS”. It may have been replaced by the USD values | corrected | Page 15  Line 10  Page 18  Line 7 |
|  | Study setting, Study population and sample size | The MDA program targeted to reach 200,000 adult population in 30 villages on Ukerewe Island, which is highly endemic for intestinal schistosomiasis. Maybe rephrase to The MDA program has a target adult population of 200,000 across 30 villages on Ukerewe Island, which is highly endemic for intestinal schistosomiasis | Changed as suggested by reviewer | Page 11  Line 1-2 |
|  | Results (Cost Projection for MDA Expansion) | Cost Projection for MDA Expansion: “The chart illustrates” Change to Figure X illustrates | corrected | Page 20  Line 3 |
|  | Results | Figure 3 – I would add comms to indicate thousands (1,000 rather than 1000 ) | Commas added | Page 21  Figure 3 |
|  | Discussion | MDA is spelled out several times in the Discussion | Corrected | Through-out the document |
|  | Conclusion | Make sure the placement of USD around the value is consistent. You generally have it before but in the conclusion, it appears after “1 USD” | Corrected | Page 26  Line 3 |
|  | Methodology | Please clarify in the text if praziquantel was donated or not – and add how it was valued (cost per tablet). | Donated by WHO and they are distributed by MoH to district health centers based on the needs and then are distributed to the nearest Health center where community drug distributors collected them from | Page 10  **Line 13 to 18** |
|  | Results | Figure 2 – The labelling of the cost inputs is inconsistent – sometimes with a capital letter but now always. I would remove the title above the figure – this is captured by the title below | The title above removed | Page 19  Figure 2 |
|  |  | This study is very key to the effort geared towards ending NTDs in Tanzania, and other endemic countries. Findings will help policy makers make informed and cost-effective decisions towards reducing the burden of NTDs. Dissemination of findings in time is therefore advised especially since the Phase II of the programs must have commenced. | So far disseminated to the Ukerewe MDA program implementers. |  |
