# Supplementary material for: Cost analysis of implementing community-based mass drug administration for schistosomiasis control among adult individuals using community drug distributors in Ukerewe district council, North-Western Tanzania
Source: PLoS Negl Trop Dis. 2026 Jul 6;20(7):e0014470. doi: 10.1371/journal.pntd.0014470 (PMC13354067; doi:10.1371/journal.pntd.0014470)
Supplement: Attachment — Submitted filename: Assessment report for PNTD-D-25-02153R1.docx [file pntd.0014470.s008.docx]

**Assessment report for the manuscript titled Cost analysis of implementing community-based mass drug administration for schistosomiasis control among adult individuals using community drug distributors in Ukerewe district council, North-Western Tanzania**

Manuscript Number: PNTD-D-25-02153R1

1. Page 3, Abstract: The objective(s) of the study should be clearly stated “*estimate the implementation costs of the first phase of the Ukerewe Schistosomiasis Control Program among the adult population residing on Ukerewe Island, Tanzania.”*

2. Page 10, Study population: Considering the fact that your study was among adult population residing in Ukerewe Island, how did you sift off or stratify the minors in the population (*The MDA program has targeted 200,000 individuals (15 years and above) across 30 villages on Ukerewe Island...)*
