# Supplementary material for: Cost analysis of implementing community-based mass drug administration for schistosomiasis control among adult individuals using community drug distributors in Ukerewe district council, North-Western Tanzania
Source: PLoS Negl Trop Dis. 2026 Jul 6;20(7):e0014470. doi: 10.1371/journal.pntd.0014470 (PMC13354067; doi:10.1371/journal.pntd.0014470)
Supplement: Attachment — Submitted filename: Response to reviewers.docx [file pntd.0014470.s010.docx]

PLOS Neglected Tropical Diseases

Dear editor,

Re: Resubmission of manuscript titled **“Cost analysis of implementing community-based mass drug administration for schistosomiasis control among adult individuals using community drug distributors in Ukerewe district council, North-Western Tanzania**”

We are grateful for the opportunity to resubmit our manuscript for consideration in your journal. We sincerely thank the reviewers for their constructive comments, which have helped us improve the content and quality of the manuscript. We believe we have addressed all the concerns to their satisfaction; however, we remain willing to undertake further revisions if deemed necessary.

Below are point by point responses to the raised comment.

**RESPONSE TO REVIEWER 2 COMMENTS**

| **S/N** | **Section** | **Comments from the reviewer** | **PI’s response** | **Page No. where changes have been made** |
| --- | --- | --- | --- | --- |
| 1 | Methods | The objective(s) of the study should reflect clearly in the abstract. The study design appropriate to address the stated objectives | Added the objective of the study in the abstract “This study aimed to estimate the implementation costs of the first phase of the Ukerewe Schistosomiasis Control Program among the adult population residing on Ukerewe Island, Tanzania.” | Page 3 |
| 2 | Methods | The population was clearly described however the methodology did not tell us how the adult data was sifted from the whole population data (15 years and above). | Note that the primary study included the population which was outside the school environment. Individual aged 15 years and above living in the study villages and out of the school. School aged children aged are treated in schools  The data included in this paper included all individuals aged 15years and above | Page 11 |
|  | Methods | The sample size was stated clearly but calculations were not shown to ensure adequate power. Statistical analysis used was appropriate to support conclusions There are no concerns about ethical or regulatory requirements | No formal sample size calculation was performed because this study employed a census approach, whereby all available expenditure and financial records related to the implementation of the MDA program during the study period were included in the analysis. | Page 11 |
|  | Editorial and Data Presentation Modifications? | 1. Page 3, Abstract: The objective(s) of the study should be clearly stated “estimate the implementation costs of the first phase of the Ukerewe Schistosomiasis Control Program among the adult population residing on Ukerewe Island, Tanzania.” | Correction done and now it read: - “This study aimed to estimate the implementation costs of the first phase of the Ukerewe Schistosomiasis Control Program among the adult population residing on Ukerewe Island, Tanzania.” | Page 3 |
| 3 | Editorial and Data Presentation Modifications? | 2. Page 10, Study population: Considering the fact that the study was conducted among adult population residing in Ukerewe Island, how did you sift off or stratify the minors (age 15 to 17 years) in the population (The MDA program has targeted 200,000 individuals (15 years and above) across 30 villages on Ukerewe Island...). | The study considered all those who were outside the school environment aged 15 years and above. Children attending schools were not included, they receive treatment within the school environment. Written informed consent from parents/guardians were obtained for the individuals aged 15-17 years plus written informed assent were obtained from all individuals aged 15-17 years. the program records and expenditure data were aggregated at the intervention level, age-specific costs were not recorded. Data for the age groups 15-17 years were pooled together with the rest of the population. | Page 11 |
