# Supplementary material for: Cost analysis of implementing community-based mass drug administration for schistosomiasis control among adult individuals using community drug distributors in Ukerewe district council, North-Western Tanzania
Source: PLoS Negl Trop Dis. 2026 Jul 6;20(7):e0014470. doi: 10.1371/journal.pntd.0014470 (PMC13354067; doi:10.1371/journal.pntd.0014470)
Supplement: Peer review history

# Peer review: Decision Letter 0

**DOI:** 10.1371/journal.pntd.0014470.r001

**Authors:** Maria Victoria Periago, Javier Gandasegui

24 Feb 2026

PNTD-D-25-02153

Cost analysis of implementing community-based mass drug administration for schistosomiasis control among adult individuals using community drug distributors in Ukerewe district council, North-Western Tanzania

PLOS Neglected Tropical Diseases

Dear Dr. Bwimba,

Thank you for submitting your manuscript to PLOS Neglected Tropical Diseases. After careful consideration, we feel that it has merit but does not fully meet PLOS Neglected Tropical Diseases's publication criteria as it currently stands. Therefore, we invite you to submit a revised version of the manuscript that addresses the points raised during the review process.

Please submit your revised manuscript within by Apr 25 2026 11:59PM. If you will need more time than this to complete your revisions, please reply to this message or contact the journal office at plosntds@plos.org. When you're ready to submit your revision, log on to https://www.editorialmanager.com/pntd/ and select the 'Submissions Needing Revision' folder to locate your manuscript file.

Please include the following items when submitting your revised manuscript:

* A letter that responds to each point raised by the editor and reviewer(s). You should upload this letter as a separate file labeled 'Response to Reviewers'. This file does not need to include responses to any formatting updates and technical items listed in the 'Journal Requirements' section below.

* A marked-up copy of your manuscript that highlights changes made to the original version. You should upload this as a separate file labeled 'Revised Manuscript with Track Changes'.

* An unmarked version of your revised paper without tracked changes. You should upload this as a separate file labeled 'Manuscript'.

If you would like to make changes to your financial disclosure, competing interests statement, or data availability statement, please make these updates within the submission form at the time of resubmission. Guidelines for resubmitting your figure files are available below the reviewer comments at the end of this letter.

We look forward to receiving your revised manuscript.

Kind regards,

Javier Gandasegui

Academic Editor

PLOS Neglected Tropical Diseases

María Periago

Section Editor

PLOS Neglected Tropical Diseases

Shaden Kamhawi

co-Editor-in-Chief

PLOS Neglected Tropical Diseases

orcid.org/0000-0003-4304-636XX

Paul Brindley

co-Editor-in-Chief

PLOS Neglected Tropical Diseases

orcid.org/0000-0003-1765-0002

**Journal Requirements:**

If the reviewer comments include a recommendation to cite specific previously published works, please review and evaluate these publications to determine whether they are relevant and should be cited. There is no requirement to cite these works unless the editor has indicated otherwise.

1) Please ensure that the CRediT author contributions listed for every co-author are completed accurately and in full.

At this stage, the following Authors/Authors require contributions: Jackson M. Bwimba, Alphoncina Kagaigai, Elizabeth Gudu, Crecencia Chiombo, Irene Msirikale, Humphrey Mazigo, Amani Thomas Mori, Charles Guya Mkombe, Saskia Kreibich, Christa Kasang, Antje Fuss, Andreas Mueller, and Ruchius Philbert. Please ensure that the full contributions of each author are acknowledged in the "Add/Edit/Remove Authors" section of our submission form.

The list of CRediT author contributions may be found here: https://journals.plos.org/plosntds/s/authorship#loc-author-contributions

2) We do not publish any copyright or trademark symbols that usually accompany proprietary names, eg ©,  ®, or TM  (e.g. next to drug or reagent names). Therefore please remove all instances of trademark/copyright symbols throughout the text, including:

- ® on page: 2.

3) Please upload all main figures as separate Figure files in .tif or .eps format. For more information about how to convert and format your figure files please see our guidelines:

https://journals.plos.org/plosntds/s/figures

4) In the online submission form, you indicated that All data supporting the findings of this study will be made available and attached during the submission process. Any additional information required by the editors or reviewers will be provided upon request.. All PLOS journals now require all data underlying the findings described in their manuscript to be freely available to other researchers, either

1. In a public repository

2. Within the manuscript itself

3. Uploaded as supplementary information.

This policy applies to all data except where public deposition would breach compliance with the protocol approved by your research ethics board. If your data cannot be made publicly available for ethical or legal reasons (e.g., public availability would compromise patient privacy), please explain your reasons by return email and your exemption request will be escalated to the editor for approval. Your exemption request will be handled independently and will not hold up the peer review process, but will need to be resolved should your manuscript be accepted for publication. One of the Editorial team will then be in touch if there are any issues.

5) Please amend your detailed Financial Disclosure statement. This is published with the article. It must therefore be completed in full sentences and contain the exact wording you wish to be published.

1) State the initials, alongside each funding source, of each author to receive each grant. For example: "This work was supported by the National Institutes of Health (####### to AM; ###### to CJ) and the National Science Foundation (###### to AM).".

If you did not receive any funding for this study, please simply state: u201cThe authors received no specific funding for this work.u201d

6) Please ensure that the funders and grant numbers match between the Financial Disclosure field and the Funding Information tab in your submission form. Note that the funders must be provided in the same order in both places as well.

**Reviewers' Comments:**

Reviewer's Responses to Questions

**Key Review Criteria Required for Acceptance?**

As you describe the new analyses required for acceptance, please consider the following:

**Methods**

-Are the objectives of the study clearly articulated with a clear testable hypothesis stated?

-Is the study design appropriate to address the stated objectives?

-Is the population clearly described and appropriate for the hypothesis being tested?

-Is the sample size sufficient to ensure adequate power to address the hypothesis being tested?

-Were correct statistical analysis used to support conclusions?

-Are there concerns about ethical or regulatory requirements being met?

Reviewer #1: (No Response)

Reviewer #2: The objectives of the study clearly articulated.

The study design appropriate to address the stated objectives

The population was not clearly described

The sample size was not stated clearly to ensure adequate power.

Statistical analysis used was appropriate to support conclusions

There are no concerns about ethical or regulatory requirements

**********

**Results**

-Does the analysis presented match the analysis plan?

-Are the results clearly and completely presented?

-Are the figures (Tables, Images) of sufficient quality for clarity?

Reviewer #1: (No Response)

Reviewer #2: The analysis presented matched the analysis plan

The results were clearly and completely presented

The figures (Tables, Images) are of sufficient quality for clarity

**********

**Conclusions**

-Are the conclusions supported by the data presented?

-Are the limitations of analysis clearly described?

-Do the authors discuss how these data can be helpful to advance our understanding of the topic under study?

-Is public health relevance addressed?

Reviewer #1: (No Response)

Reviewer #2: The conclusions are supported by the data presented

No limitations was described for analysis

The authors discuss how these data can be helpful to advance our understanding of the topic under study

Public health relevance addressed

**********

**Editorial and Data Presentation Modifications?**

Use this section for editorial suggestions as well as relatively minor modifications of existing data that would enhance clarity. If the only modifications needed are minor and/or editorial, you may wish to recommend “Minor Revision” or “Accept”.

Reviewer #1: (No Response)

Reviewer #2: MINOR REVISION

1. Page 3, Author Summary, line 4: insert space between adultssuch

2. page 5, Introduction, line 14: insert space between lowincome

3. page 5, Introduction, line 18: insert space between highrisk

4. page 8, Cost approach, line 4: insert hyphen between communitybased

5. page 8, Cost approach, line 6: correct typo susch

6. page 8, Study setting, line 2: Why not use a more recent census estimate or projection since the growth rate is available? This might show the true difference in the number of people reached (received praziquantel) in 2025 (310,807 2022 census versus 387,815 that were reached at the end of 2025). Study population and sample size not clearly stated.

7. Page 10, Cost data Collection, line 18: Correct typo methothod

8. Page 11, Data Management, line 8 and 12: insert space between inflationadjusted and scaleup

9. Page 12, Treatment Coverage, line 1: who exactly are the target of the MDA programme? All the community members or Non-school children?

10. Page 12, Treatment Coverage, line 6: What was the extent of the expansion? Was the expansion within Ukerewe district/ Island/ MDA programme?

11. Page 12, Cost profile for the first phase of MDA, line 7: Judging by Table 1, the cost by year is not TZS USD 80,502

12. Page 14, Cost associated with each MDA activity, line 4: Judging by Table 1, the cost by year is not TZS USD 27,770

13. Page 15, Cost projection for MDA expansion, line 2: The District 2022 census estimate stated under study setting (387,815) is different from the one stated here (202,389). The two should have been stated and clarified under the study setting.

14. Page 17, Discussion, line 27 and 37: insert space between costefficiency

**********

**Summary and General Comments**

Use this section to provide overall comments, discuss strengths/weaknesses of the study, novelty, significance, general execution and scholarship. You may also include additional comments for the author, including concerns about dual publication, research ethics, or publication ethics. If requesting major revision, please articulate the new experiments that are needed.

Reviewer #1: This is an interesting cost analysis of MDA in Tanzania. Having such cost data published is vital for informing our understanding of how to optimize treatment for schistosomiasis.

I have the following comments and suggestions;

My most important comments relates to economic vs financial costs. You mention that both were collected in the methods but that is all. I believe that both financial and economic costs should be clearly presented and that further information in the methods is needed to explain how you estimated the opportunity cost of resources that did not have a budget line item or which were donated?

For Capital costs. Straight line depreciation method is appropriate for financial costs but not appropriate for economic costs. See the following paper for further guidance: https://doi.org/10.1093/heapol/17.1.112

In the introduction, you refer to “The initiative currently operates across 30 sentinel villages”. Apologies if this is my misunderstanding, but is this referring to an initiative that falls under the Tanzania SCH program, or the Ukerewe Schistosomiasis Control Program? The current wording implies that it is the whole program, as Ukerewe is not really mentioned until the end of the paragraph, and Tanzania as a whole is what is talked about in the preceding paragraph. Please clarify and modify the text accordingly. Related to this, having more background on the Ukerewe MDA program in the introduction would be beneficial, such as whether it only targets adults or SAC as well?

The order of the methods can be hard to follow. I suggest adding what you have under the Time horizon subsection to the Study design and perspective subsection.

The phase non-school children can be confusing in this context. It is a reference to adult children not in school. More generally, the age groups treated need further clarification.

Discussion – It would be helpful to have a few sentences comparing the magnitude of the MDA cost found ($1.66 per treatment) to other MDA cost estimates in more detail (particularly in Tanzania). The benchmark of $0.5 is still used but could be out of date.

Please clarify how the drugs were distributed – community volunteers or health workers ? If community volunteers how where they valued.

The authors should consider a limitations section.

Minor comments:

• Abstract methods - there's an extra space in front of one of the full stops.

• In several places, there is a miss space before a reference bracket e.g. Page 5 – Missing space before [11] “areas[11]”.

• Page 6: mass drug administration is spelled out again at the beginning of second paragraph. Can just use MDA here

• Methods “provider perspective, capturing all the expenses of resources consumed inimplementing MDA activities”. Saying “capturing all the expenses of resources” .. sounds more like the societal perspective” I would modify the text to make the implications of the perspective clearer.

• The MDA program targeted to reach 200,000 adult population in 30 villages on Ukerewe Island, which is highly endemic for intestinal schistosomiasis. Maybe rephrase to The MDA program has a target adult population of 200,000 across 30 villages on Ukerewe Island, which is highly endemic for intestinal schistosomiasis

• Page 10 methothod typo (method)

• Results Cost associated with each MDA activity. Delete (M&E) as you do not use it. Check why you have the “TZS”. It may have been replaced by the USD values

• Results Cost Projection for MDA Expansion: “The chart illustrates” Change to Figure X illustrates

• Figure 3 – I would add comms to indicate thousands (1,000 rather than 1000 )

• MDA is spelled out several times in the Discussion

• Make sure the placement of USD around the value is consistent. You generally have it before but in the conclusion, it appears after “1 USD”

• Please clarify in the text if praziquantel was donated or not – and add how it was valued (cost per tablet).

• Figure 2 – The labelling of the cost inputs is inconsistent – sometimes with a capital letter but now always. I would remove the title above the figure – this is captured by the title below

Reviewer #2: This study is very key to the effort geared towards ending NTDs in Tanzania, and other endemic countries. Findings will help policy makers make informed and cost effective decisions towards reducing the burden of NTDs. Dissemination of findings in time is therefore advised especially since the Phase II of the programs must have commenced.

**********

PLOS authors have the option to publish the peer review history of their article (what does this mean?). If published, this will include your full peer review and any attached files.

If you choose “no”, your identity will remain anonymous but your review may still be made public.

**Do you want your identity to be public for this peer review?**  For information about this choice, including consent withdrawal, please see our Privacy Policy.

Reviewer #1: **Yes:** Hugo Turner

Reviewer #2: No

[NOTE: If reviewer comments were submitted as an attachment file, they will be attached to this email and accessible via the submission site. Please log into your account, locate the manuscript record, and check for the action link "View Attachments". If this link does not appear, there are no attachment files.]

**Figure resubmission:**

While revising your submission, we strongly recommend that you use PLOS’s NAAS tool (https://ngplosjournals.pagemajik.ai/artanalysis) to test your figure files. NAAS can convert your figure files to the TIFF file type and meet basic requirements (such as print size, resolution), or provide you with a report on issues that do not meet our requirements and that NAAS cannot fix.

After uploading your figures to PLOS’s NAAS tool - https://ngplosjournals.pagemajik.ai/artanalysis, NAAS will process the files provided and display the results in the "Uploaded Files" section of the page as the processing is complete. If the uploaded figures meet our requirements (or NAAS is able to fix the files to meet our requirements), the figure will be marked as "fixed" above. If NAAS is unable to fix the files, a red "failed" label will appear above. When NAAS has confirmed that the figure files meet our requirements, please download the file via the download option, and include these NAAS processed figure files when submitting your revised manuscript.

**Reproducibility:**

To enhance the reproducibility of your results, we recommend that authors of applicable studies deposit laboratory protocols in protocols.io, where a protocol can be assigned its own identifier (DOI) such that it can be cited independently in the future. Additionally, PLOS ONE offers an option to publish peer-reviewed clinical study protocols. Read more information on sharing protocols at https://plos.org/protocols?utm_medium=editorial-email&utm_source=authorletters&utm_campaign=protocols

Attachment Submitted filename: Assessment report for the manuscript PNTD-D-25-02153.docx

# Author comment: Author response to Decision Letter 1

**DOI:** 10.1371/journal.pntd.0014470.r002

28 Apr 2026

Attachment Submitted filename: Response to reviewers .docx

# Peer review: Decision Letter 1

**DOI:** 10.1371/journal.pntd.0014470.r003

**Authors:** Maria Victoria Periago, Javier Gandasegui, Maria Victoria Periago, Javier Gandasegui

26 May 2026

PNTD-D-25-02153R1

Cost analysis of implementing community-based mass drug administration for schistosomiasis control among adult individuals using community drug distributors in Ukerewe district council, North-Western Tanzania

PLOS Neglected Tropical Diseases

Dear Dr. Bwimba,

Thank you for submitting your manuscript to PLOS Neglected Tropical Diseases. After careful consideration, we feel that it has merit but does not fully meet PLOS Neglected Tropical Diseases's publication criteria as it currently stands. Therefore, we invite you to submit a revised version of the manuscript that addresses the points raised during the review process.

Please submit your revised manuscript by Jun 25 2026 11:59PM. If you will need more time than this to complete your revisions, please reply to this message or contact the journal office at plosntds@plos.org. When you're ready to submit your revision, log on to https://www.editorialmanager.com/pntd/ and select the 'Submissions Needing Revision' folder to locate your manuscript file.

Please include the following items when submitting your revised manuscript:* A letter that responds to each point raised by the editor and reviewer(s). You should upload this letter as a separate file labeled 'Response to Reviewers'. This file does not need to include responses to any formatting updates and technical items listed in the 'Journal Requirements' section below.* A marked-up copy of your manuscript that highlights changes made to the original version. You should upload this as a separate file labeled 'Revised Manuscript with Track Changes'.* An unmarked version of your revised paper without tracked changes. You should upload this as a separate file labeled 'Manuscript'.If you would like to make changes to your financial disclosure, competing interests statement, or data availability statement, please make these updates within the submission form at the time of resubmission. Guidelines for resubmitting your figure files are available below the reviewer comments at the end of this letter.As the corresponding author, your ORCID iD is verified in the submission system and will appear in the published article. PLOS supports the use of ORCID, and we encourage all coauthors to register for an ORCID iD and use it as well. Please encourage your coauthors to verify their ORCID iD within the submission system before final acceptance, as unverified ORCID iDs will not appear in the published article. Only the individual author can complete the verification step; PLOS staff cannot verify ORCID iDs on behalf of authors.We look forward to receiving your revised manuscript.

Kind regards,

Javier Gandasegui

Academic Editor

PLOS Neglected Tropical Diseases

María Periago

Section Editor

PLOS Neglected Tropical Diseases

Shaden Kamhawi

co-Editor-in-Chief

PLOS Neglected Tropical Diseases

orcid.org/0000-0003-4304-636XX

Paul Brindley

co-Editor-in-Chief

PLOS Neglected Tropical Diseases

orcid.org/0000-0003-1765-0002

**Journal Requirements:**

If the reviewer comments include a recommendation to cite specific previously published works, please review and evaluate these publications to determine whether they are relevant and should be cited. There is no requirement to cite these works unless the editor has indicated otherwise.

1) Your manuscript's sections are not in the correct order.  Please amend to the following order: Abstract, Introduction, Results, Discussion, and Methods

2) Thank you for including an Ethics Statement for your study. Please include:

i) A statement that formal consent was obtained (must state whether verbal/written) OR the reason consent was not obtained (e.g. anonymity). NOTE: If child participants, the statement must declare that formal consent was obtained from the parent/guardian.].

3) Please amend your detailed Financial Disclosure statement. This is published with the article. It must therefore be completed in full sentences and contain the exact wording you wish to be published.

1) State what role the funders took in the study. If the funders had no role in your study, please state: "The funders had no role in study design, data collection and analysis, decision to publish, or preparation of the manuscript."

2) If any authors received a salary from any of your funders, please state which authors and which funders..

If you did not receive any funding for this study, please simply state: u201cThe authors received no specific funding for this work.u201d

**Reviewers' comments:**

Reviewer's Responses to Questions

**Key Review Criteria Required for Acceptance?**

As you describe the new analyses required for acceptance, please consider the following:

**Methods**

-Are the objectives of the study clearly articulated with a clear testable hypothesis stated?

-Is the study design appropriate to address the stated objectives?

-Is the population clearly described and appropriate for the hypothesis being tested?

-Is the sample size sufficient to ensure adequate power to address the hypothesis being tested?

-Were correct statistical analysis used to support conclusions?

-Are there concerns about ethical or regulatory requirements being met?

Reviewer #1: (No Response)

Reviewer #2: The objective(s) of the study should reflect clearly in the abstract.

The study design appropriate to address the stated objectives

The population was clearly described however the methodology did not tell us how the adult data was sifted from the whole population data (15 years and above)

The sample size was stated clearly but calculations were not shown to ensure adequate power.

Statistical analysis used was appropriate to support conclusions

There are no concerns about ethical or regulatory requirements

**********

**Results**

-Does the analysis presented match the analysis plan?

-Are the results clearly and completely presented?

-Are the figures (Tables, Images) of sufficient quality for clarity?

Reviewer #1: (No Response)

Reviewer #2: The analysis presented matched the analysis plan

The results were clearly and completely presented

The figures (Tables, Images) are of sufficient quality for clarity

**********

**Conclusions**

-Are the conclusions supported by the data presented?

-Are the limitations of analysis clearly described?

-Do the authors discuss how these data can be helpful to advance our understanding of the topic under study?

-Is public health relevance addressed?

Reviewer #1: (No Response)

Reviewer #2: The conclusions are supported by the data presented

No limitations was described for analysis

The authors discuss how these data can be helpful to advance our understanding of the topic under study

Public health relevance addressed

**********

**Editorial and Data Presentation Modifications?**

Use this section for editorial suggestions as well as relatively minor modifications of existing data that would enhance clarity. If the only modifications needed are minor and/or editorial, you may wish to recommend “Minor Revision” or “Accept”.

Reviewer #1: (No Response)

Reviewer #2: MINOR REVISION

1. Page 3, Abstract: The objective(s) of the study should be clearly stated “estimate the implementation costs of the first phase of the Ukerewe Schistosomiasis Control Program among the adult population residing on Ukerewe Island, Tanzania.”

2. Page 10, Study population: Considering the fact that the study was conducted among adult population residing in Ukerewe Island, how did you sift off or stratify the minors (age 15 to 17 years) in the population (The MDA program has targeted 200,000 individuals (15 years and above) across 30 villages on Ukerewe Island...)

**********

**Summary and General Comments**

Use this section to provide overall comments, discuss strengths/weaknesses of the study, novelty, significance, general execution and scholarship. You may also include additional comments for the author, including concerns about dual publication, research ethics, or publication ethics. If requesting major revision, please articulate the new experiments that are needed.

Reviewer #1: (No Response)

Reviewer #2: This study is very key to the effort geared towards ending NTDs in Tanzania, and other endemic countries. Findings will help policy makers make informed and cost effective decisions towards reducing the burden of NTDs. Dissemination of findings on time is therefore advised especially since the Phase II of the programs must have commenced.

**********

PLOS authors have the option to publish the peer review history of their article (what does this mean?). If published, this will include your full peer review and any attached files.

If you choose “no”, your identity will remain anonymous but your review may still be made public.

**Do you want your identity to be public for this peer review?**  For information about this choice, including consent withdrawal, please see our Privacy Policy.

Reviewer #1: No

Reviewer #2: No

[NOTE: If reviewer comments were submitted as an attachment file, they will be attached to this email and accessible via the submission site. Please log into your account, locate the manuscript record, and check for the action link "View Attachments". If this link does not appear, there are no attachment files.]**Figure resubmission:**

While revising your submission, we strongly recommend that you use PLOS’s NAAS tool (https://ngplosjournals.pagemajik.ai/artanalysis) to test your figure files. NAAS can convert your figure files to the TIFF file type and meet basic requirements (such as print size, resolution), or provide you with a report on issues that do not meet our requirements and that NAAS cannot fix.

After uploading your figures to PLOS’s NAAS tool - https://ngplosjournals.pagemajik.ai/artanalysis, NAAS will process the files provided and display the results in the "Uploaded Files" section of the page as the processing is complete. If the uploaded figures meet our requirements (or NAAS is able to fix the files to meet our requirements), the figure will be marked as "fixed" above. If NAAS is unable to fix the files, a red "failed" label will appear above. When NAAS has confirmed that the figure files meet our requirements, please download the file via the download option, and include these NAAS processed figure files when submitting your revised manuscript.**Reproducibility:** To enhance the reproducibility of your results, we recommend that authors of applicable studies deposit laboratory protocols in protocols.io, where a protocol can be assigned its own identifier (DOI) such that it can be cited independently in the future. Additionally, PLOS ONE offers an option to publish peer-reviewed clinical study protocols. Read more information on sharing protocols at https://plos.org/protocols?utm_medium=editorial-email&utm_source=authorletters&utm_campaign=protocols

Attachment Submitted filename: Assessment report for PNTD-D-25-02153R1.docx

# Author comment: Author response to Decision Letter 2

**DOI:** 10.1371/journal.pntd.0014470.r004

11 Jun 2026

Attachment Submitted filename: Response to reviewers.docx

# Editor report: Decision Letter 2

**DOI:** 10.1371/journal.pntd.0014470.r005

**Authors:** Maria Victoria Periago, Javier Gandasegui, Maria Victoria Periago, Javier Gandasegui, Maria Victoria Periago, Javier Gandasegui

15 Jun 2026

Dear Pharm. Bwimba,

We are pleased to inform you that your manuscript 'Cost analysis of implementing community-based mass drug administration for schistosomiasis control among adult individuals using community drug distributors in Ukerewe district council, North-Western Tanzania' has been provisionally accepted for publication in PLOS Neglected Tropical Diseases.

Before your manuscript can be formally accepted you will need to complete some formatting changes, which you will receive in a follow up email. A member of our team will be in touch with a set of requests.

Please note that your manuscript will not be scheduled for publication until you have made the required changes, so a swift response is appreciated.

IMPORTANT: The editorial review process is now complete. PLOS will only permit corrections to spelling, formatting or significant scientific errors from this point onwards. Requests for major changes, or any which affect the scientific understanding of your work, will cause delays to the publication date of your manuscript.

Should you, your institution's press office or the journal office choose to press release your paper, you will automatically be opted out of early publication. We ask that you notify us now if you or your institution is planning to press release the article. All press must be co-ordinated with PLOS.

Thank you again for supporting Open Access publishing; we are looking forward to publishing your work in PLOS Neglected Tropical Diseases.

Best regards,

Javier Gandasegui

Academic Editor

PLOS Neglected Tropical Diseases

Maria Victoria Periago

Section Editor

PLOS Neglected Tropical Diseases

Shaden Kamhawi

co-Editor-in-Chief

PLOS Neglected Tropical Diseases

orcid.org/0000-0003-4304-636XX

Paul Brindley

co-Editor-in-Chief

PLOS Neglected Tropical Diseases

orcid.org/0000-0003-1765-0002

***********************************************************

# Editor report: Acceptance letter

**DOI:** 10.1371/journal.pntd.0014470.r006

**Authors:** Maria Victoria Periago, Javier Gandasegui, Maria Victoria Periago, Javier Gandasegui, Maria Victoria Periago, Javier Gandasegui

Dear Pharm. Bwimba,

We are delighted to inform you that your manuscript, "Cost analysis of implementing community-based mass drug administration for schistosomiasis control among adult individuals using community drug distributors in Ukerewe district council, North-Western Tanzania," has been formally accepted for publication in PLOS Neglected Tropical Diseases.

We have now passed your article onto the PLOS Production Department who will complete the rest of the publication process. All authors will receive a confirmation email upon publication.

The corresponding author will soon be receiving a typeset proof for review, to ensure errors have not been introduced during production. Please review the PDF proof of your manuscript carefully, as this is the last chance to correct any scientific or type-setting errors. Please note that major changes, or those which affect the scientific understanding of the work, will likely cause delays to the publication date of your manuscript. Note: Proofs for Front Matter articles (Editorial, Viewpoint, Symposium, Review, etc...) are generated on a different schedule and may not be made available as quickly.

Soon after your final files are uploaded, the early version of your manuscript will be published online unless you opted out of this process. The date of the early version will be your article's publication date. The final article will be published to the same URL, and all versions of the paper will be accessible to readers.

For Research Articles, you will receive an invoice from PLOS for your publication fee after your manuscript has reached the completed accept phase. If you receive an email requesting payment before acceptance or for any other service, this may be a phishing scheme. Learn how to identify phishing emails and protect your accounts at https://explore.plos.org/phishing.

Thank you again for supporting open-access publishing; we are looking forward to publishing your work in PLOS Neglected Tropical Diseases.

Best regards,

Shaden Kamhawi

co-Editor-in-Chief

PLOS Neglected Tropical Diseases

Paul Brindley

co-Editor-in-Chief

PLOS Neglected Tropical Diseases
